# Struggling in pandemic times: Migrant women's virtual political organization during the COVID‐19 crisis in Spain

**DOI:** 10.1111/imig.13034

**Published:** 2022-07-20

**Authors:** Emma Martín‐Díaz, Simone Castellani

**Affiliations:** ^1^ Universidad de Sevilla Sevilla Spain; ^2^ Universidad de Cádiz, Jerez de la Frontera, Spain & CIES‐Iscte Lisbon Portugal

## Abstract

During the COVID‐19 pandemic in Spain, the lockdowns brought about the loss of labour and social rights for many migrant women working in highly informal employment sectors. Drawing on digital ethnography, this article examines the role of migrant women in political mobilization within migrant associations during 2020 in Spain, when online interactions assumed primary importance. First, it shows how migrant organizations create bricolage by combining economic, social, cultural and political resources to organize migrants in Spain during COVID‐19 within the digital space. Second, the article highlights how the reactions of the migrant population to the pandemic in Spain represent an act of disruption led mainly by women, within the dynamics of social conflict and political activism that characterize migratory processes. This analysis displays as well the crucial role played by care relationships in these contexts of crises.

## INTRODUCTION

During the COVID‐19 pandemic in Spain, highly informal work sectors such as domestic and care work, as well as hospitality and agriculture, with a high presence of migrant women workers, were strongly hit, especially during the periods of strict lockdown ordered by the government.^1^ Public enforcement controls were maximal, making it almost impossible for the informal economy to function. Women were highly affected, having to renounce their jobs or being fired for taking care of the children after the school closures and compulsory home schooling (Hernández Cordero et al., 2021; Lagomarsino et al., 2020). Those who worked as domestic and care assistants were banned by employers out of fear of contagion or forced to cohabit (de Diego‐Cordero et al., 2021; Parella Rubio, 2021).

For many migrant households, COVID‐19 meant the loss of part (or all) of their income, which led to increasing difficulties in paying the rent and providing for dependents both in origin and destination countries (EMN/OECD, 2020). Many people with temporary residence status also lost their social rights, such as entitlement to public subsidies, because these were attached to their work and legal status (EMN/OECD, 2021). For them, the pandemic represented a deterioration in economic and working conditions, as well as their social rights (EMN/OECD, 2021).

In Spain, temporary permits were extended for regular migrants to respond to this situation. Nevertheless, despite protests by some sectors of civil society calling for a general regularization of migrants with irregular administrative status, the government discarded it, leaving de facto people in the most precarious situation on the verge of social exclusion. First, they had no guarantee of health coverage in the context of a pandemic, even if some Autonomous Communities alleviated this by intermittently allowing access to the public health system for all irregular residents during the “state of alarm” (Perna & Moreno Fuentes, 2021). Moreover, it became impossible for many irregular migrants working informally to access labour and social programmes, such as the minimum vital income or the temporary lay‐offs, which were the primary protection measures during the pandemic.

At the beginning of the first lockdown, in the spring of 2020, a coordinated campaign^2^ started, boosted by migrant groups and supported by Spanish civil society in different parts of the country, denouncing the precariousness of migrant households and resulting in the constitution of the #*RegularizacionYa* (*#RegularizationNow*) initiative. Very quickly, this platform gained the support of 1500 organizations, including local and migrant associations, political parties and radical left unions, feminist organizations and anti‐racist groups. Together, they supported a broad‐spectrum programme centred on the following points: (1) a one‐off process of regularization for all migrants in Spain; (2) the immediate positive resolution of all asylum and refugee applications; (3) the immediate release of all persons held in centres for temporary residence (CETI) and detention centres for immigrants (CIE), with the definitive closure of these centres.

In this mobilization, the role of migrant women has stood out. They organized virtual meetings, campaigned in Online Social Networks (OSNs) and set up emergency help for people facing economic difficulties in the pandemic. Migrants themselves promoted these activities, often independently from the pro‐migrant NGOs, which had always played a central role in organizing migrant claims in the Spanish public space (Cuberos Gallardo, 2009).

This article is based on observations made of the political mobilization of migrant and pro‐migrant associations, formal and informal, within the digital space^3^ (Góralska, 2020) during the first general lockdown in Spain (March–June 2020). We pay special attention to the organizational practices of migrant associations, particularly those integrated by migrant women speaking out and mobilizing politically (in‐ and outside the digital space) at the beginning of the pandemic. The aim is, first, to analyse migrant organizations' creative bricolage (Phillimore et al., 2019) with the resources (economic, social, cultural and political) at their disposal through virtual performances and social networks. This organizational bricolage is understood as a way for migrants to display their structural vulnerable condition (Holmes, 2011) and, at the same time, fight against it claiming for regularization and full access to labour and social rights. Second, the purpose is to evaluate the pandemic as a shock that let the door open for a reaction from migrant organizations that can be understood as a creative break (Isin & Nielsen, 2008) and an act of disruption (Swerts & Nicholls, 2021) within the consolidated practices and habits of migrant political activism in Spain. Besides, the article investigates the crucial role of care relationships in generating alternative forms of migrant organizations seeking political transformation (Stock, 2019). Finally, it displays how, during COVID‐19, the substantial dependency of migrant organizations on the modes and forms of protest of pro‐rights associations (anti‐racist NGOs, feminist activists and labour unions) was put into question, enabling them to find a common, interdependent, autonomous voice.

## THEORETICAL FRAMEWORK

In the Global North, the dynamics of global capitalism have intensified employer demands for international migrant workers in specific sectors such as the care and domestic industries, where migrant women are over‐represented, leading to the well‐studied phenomenon of global circulation of care (Anderson, 2000; Sassen, 2000; Williams, 2011 among others). The care and domestic labour sectors, as well as hospitality, logistics and agriculture, where migrants are also primarily represented, are characterized by high levels of precariousness and informality (Kofman, 2003), constituting a considerable portion of the secondary labour market (Berger & Piore, 1980). The increasing dualism of labour markets also stratifies access to welfare, meaning a progressive loss of social rights for a significant amount of the population, both native and migrant, working in jobs in this secondary labour market (Hausermann & Schwander, 2012).

During an unexpected and crushing situation like the COVID‐19 pandemic, a lack of universal safety nets, for instance a universal health system or a basic income, meant that many households that in normal times stay afloat relying on precarious and informal jobs, sank into economic and social exclusion. In fact, for many international migrants in the European Union (EU), COVID‐19 has meant losing their residence permit and, consequently, access to social assistance and services (EMN/OECD, 2021), falling into invisibility.

As Faist (2019) underlined, while in the late nineteenth century, inequality was more determined by class belonging, in the twenty‐first century, “location” (country of living) becomes the decisive determinant of inequality at the global level. Moreover, in the last half‐century, other dimensions than class, such as gender and ethnicity/race, have been central to explaining disparities in life chances within the societies. These variations caused a change within the collective actors struggling for social equality. Unions and mass parties, which represented the claims of the working class, have been increasingly replaced by a galaxy of civil society organizations that, in the last decades, have fought for the right to citizenship and against the invisibility of “no‐citizens” (women, LGBTQ+ (lesbian, gay, bisexual, transgender, queer (or questioning) and others) people, internal and migrant minorities).

The key advocacy role of civil society organizations fighting for migrant access to fundamental rights in the globalization era has been broadly documented and analysed from a critical lens (Cuberos Gallardo, 2009; Pojmann, 2008; Rother & Steinhilper, 2019; Uitermark & Nicholls, 2014). Moreover, many studies show how migrants themselves take the initiative and get involved directly in political practices and organizations, making links with parties, trade unions and NGOs (Bermúdez et al., 2014; Pennix & Rosbland, 2000), as well as replicating practices of action from past and present groups (Escudero, 2020) both in their countries of origin and destination. As actors within transnational social spaces, where not only people but also goods, services, money, ideas and practices circulate (Levitt & Schiller, 2004), they manage to create discourses and practices that overcome the national conflict frame. Transnational political activism has also been at the centre of scholarly work highlighting diaspora's crucial role in the politics of their home countries (Bermúdez et al., 2017; Martiniello & Lafleur, 2008).

Within studies of labour and industrial relations, scholars have underlined how migrant workers demonstrate their willingness to organize and improve their lives through collective action (Martín‐Díaz & Roca, 2021; Milkman, 2006), joining unions or shaping community‐based organizations (“community unionism”) (Wills & Simms, 2004). The aim was to highlight the importance of the racial or ethnic logic that underlies the particular situation of migrant workers as non‐traditional employment actors due to their over‐representation in the secondary labour market (Holgate, 2021), requiring specific trade union policies to represent their interests both in the labour space and in the communities (Roca, 2020). This literature showed that migrants’ need to create collective responses against exploitation and precariousness without the traditional tools of national unions favoured the emergence of new networks using highly experimental and hybrid forms of activism (Janine et al., 2018). This allowed them to be classified as “new social movements” (Della Porta & Tarrow, 2005). Furthermore, the relationship between labour unions and community groups is often labelled as dynamic and contradictory. Both types of organizations can have very different structural features, ideological orientations and identities, responding to different constituencies. They can cooperate for mutual benefit or shared goals, but they can also compete for resources, particular interests and hegemony (Roca & Díaz Parra, 2017; Tapia, 2013).

These studies look at the broader relationship between pro‐migrant and migrant associations in the political field. To look into migrant women political and organizational practices during the pandemic, it is useful as well to include the notion of bricolage understood as the ability to find collaborative solutions to challenging situations, combining available resources (economic, social, cultural, political) and adapting them to a particular socio‐historical context (Castellani & Roca, 2022). Organizational bricolage is a process that generates knowledge and practices that counter the taken‐for‐granted premises and have the potential of political transformation. In this sense, bricolage could be considered a tool for migrant associations' strategies to fight against the everyday inequalities they face. Migrants undertake processes of bricolage harnessing creatively different types of resources at their disposal (Levitt et al., 2017) in diverse ways within specific local and transnational networks and organizations, especially when contexts change drastically, as in the case of an economic crisis or the pandemic.

Furthermore, to analyse the political organization of women it is crucial to take into account care relationships, understanding them as an alternative form of organizing people and political transformation. Following Isin and Nielsen (2008) and Puig de la Bellacasa (2012), Stock (2019: p. 132) defines care relationships as inherent acts of citizenship because they pursue “alternate ways of engaging with the state, public institutions, or friends and family” and “they are not necessarily related to formalised and ritualised expressions of formal citizenship such as voting or protesting”. These care activities creatively break with other common sense practices that respond to social positions in terms of gender, ethnicity, class and citizenship, “simultaneously responding to this crisis with an invention, a new way of reacting to difference and injustice” (Stock, 2019: 132), transforming informal and private practices into public and political acts.

## METHODOLOGY

This paper draws upon a study carried out in the digital space during the first lockdown in Spain (15/03/2020–21/06/2020) with migrant and pro‐migrant associations, formal and informal^4^ (*n* = 12), and one civil society initiative, the platform #*RegularizacionYa* (@RegularizacionYa). These OSNs were the primary means of action and communication during the period of confinement and continue to be the main communication channel in 2022. The selection of these entities was based on the following criteria: (1) presence on OSNs (before and after the lockdowns); (2) intensity of online activity; and (3) frequency and intensity of interactions among associations. All the associations were informed that their activities on the OSNs (Twitter, Instagram and Facebook) were being monitored for research purposes, and consent was obtained via Twitter DM (direct messages). Among the 12 entities, six were analysed using observation techniques, participating in the tweets with comments and re‐tweeting the actions, and with the other six we established a collaborative ethnography (Rappaport, 2008) participating in online activities organized by them and related groups and inviting them to participate in activities organized by the ‐MIGRASCAPE project. Permission to use material from Telegram or WhatsApp was neither sought nor, therefore, obtained for reasons of confidentiality. However, they were informed about the study and reassured that the information obtained from these channels would not be used.

For 3 months, digital ethnography (Burrell, 2009; Góralska, 2020; Hine, 2012) was carried out on the OSNs of the associations. We understand the digital space both as a “culture”, a place where social interactions based on shared communication codes take place, and as a “cultural artefact”, taking into account its character as a social construct and its “context of use” (Hine, 2012). This allows for the boundary between online and offline activities to be relativized. The online activities of these organizations were followed on their Twitter and Instagram accounts. Twitter was one of the most used OSNs to communicate, promote, campaign and organize themselves. During March–June 2020, more than 3000 Twitter interactions (tweet, tweet chain, re‐tweet) were recorded. A database was set up and the interaction fields were defined based on the online and offline experiences of the actors themselves, not as dissociated realities but as interconnected spaces and temporalities.

The units of observation are the political practices and organization of these formal and informal associations in the digital space: generating debate, creating space‐times of discussion and campaigning. Special attention was given to the activities of women who stood out through their engagement within these migrant organizations during the pandemic. We had prior knowledge of the majority of these associations, which we had followed in past ethnographic researches (Castellani & Martín‐Díaz, 2019; Martín Díaz et al., 2012; Martín‐Díaz, 2012) about agricultural labour markets, domestic service and prostitution. Thus, we were aware of their previous work focused on trade union activities (denouncing working conditions, fighting for labour rights) and political activism (around the effects of the Law on Foreigners, demands for regularization), which allowed us to identify qualitative changes in terms of their internal political organization and in their relations with institutions and the rest of civil society.

The ethnographic material collected in the digital space was catalogued in digital and physical diaries, and it was analysed through a combination of critical discourse analysis (Van Dijk, 1993; Wodak & Boukala, 2015), and the ethnographic analysis of the field of relations among the subjects of the study that was shaped by a common and multi‐situated imagery. These relations (instrumental, political, of affinity or solidarity) aim to generate counter‐hegemonic discourses and practices against class, ethnic and gender inequality in the analogical and digital space as well as promoting the empowerment of migrant subjects.

One of the most evident limitations of the study is the difficulty in measuring the repercussions of online interactions in offline activities. For instance, campaigns aimed at turning a hashtag into a trending topic may represent a distorted image of the social impact of the message. On the other hand, the fact that a discourse presents a high degree of assertiveness and social denunciation can generate an image of a collective that does not reflect the practices or ideology of the whole membership. The immediacy of communication in the OSNs, such as Twitter or Instagram, has the advantage of greater participation but it is not a media prone to reflection. However, studying OSNs themselves as a unit of analysis is vital to recognize the communicative strategies, the alliances and the problems of the migrant (and non‐migrant) population during the pandemic.

## MIGRANT ASSOCIATIONS TAKE THE LEAD

Since 2008, the priorities of the European immigration agenda have shifted from the integration of migrants to border control (Hess & Kasparek, 2017). This shift is particularly noticeable in the wake of the so‐called “Arab Spring” (Aris Escarcena, 2022). Furthermore, the economic and political transformations on a global scale have led to an alteration in migratory quotas. The migratory profile and routes of social insertion are changing, as political asylum seekers now outnumber economic migrants. However, this difference is difficult to define in practice, considering that most of those who arrived in the 2010s are part of the irregular migrant flows. At present, it is estimated that more than half a million migrants in Spain are in an irregular administrative situation (Fanjul & Gálvez‐Iniesta, 2020).

COVID‐19 has brought a fundamental transformation in the organizational strategies of migrants in Spain. In a context where mobility was restricted, as during the first lockdown, only part of the migrant population kept their jobs because they worked in activities considered “essential” (e.g. health‐related jobs).^5^ However, a considerable part of this population, particularly those in an irregular administrative situation, who worked in the informal economy lost their jobs. The impossibility of moving within cities without a document proving employment placed these people in a position of vulnerability. It also generated cases of abuse, in which live‐in domestic workers were forced to choose between being locked up in the houses in which they worked or losing their jobs^6^ (de Diego‐Cordero et al., 2021; Parella Rubio, 2021).

The spread of the virus caused a wave of panic in the entrepreneurial world. The introduction of a public‐supported system of temporary lay‐offs of staff (ERTE), which relieved employers of the wage costs of their workers, was the immediate solution applied in the formal labour market. However, these measures left out all the people who work in the informal economy, where there is a clear over‐representation of migrant women in an irregular administrative situation (Fanjul & Gálvez‐Iniesta, 2020). Moreover, because of their social and labour invisibility, they did not have the right to apply for essential benefits such as the Minimum Vital Income (IMV) implemented by the Spanish government in response to the crisis brought by the pandemic.

The lockdown affected free movement and produced a decline in productivity for non‐essential economic sectors. Furthermore, it also led to a slowdown in administrative procedures in legalization processes or asylum requests, generating real emergencies among undocumented and temporary documented migrants. This situation provoked an immediate response from migrant associations, which took advantage of the opportunities offered by online social media to organize themselves into a network to disseminate their proposals and demands and set up a wide range of activities: talks, mutual support networks, denunciations of migrants’ labour situation (through workshops, conferences and videos), and significant political participation in the demonstrations against racism that took place in different cities in Spain once the lockdown measures were relaxed.

The increasing virtual presence of these associations in the pandemic context can be easily detected from observations of the OSNs. Between March and June 2020, more than 3000 tweets were launched. These numbers marked an astonishing increase in Twitter interactions from these organizations compared to December 2019–February 2020. As a matter of fact, several associations created their Twitter account during the pandemic, for instance @Jornaleras en Lucha, @Valiente Bangla, @CnaaB and the national action @regularizacionYa.

The six previous processes of migrant regularization promoted in Spain (from 1986 to 2005) resulted from campaigns in which both the State and civil society participated. Regularization generally took place when there was the perception that the presence of a significant amount of undocumented people meant more risks than benefits for society as a whole.^7^ Nevertheless, both from an institutional point of view and that of specific sectors of society, generally represented by conservative parties, a “pure” universal regularization has been presented as an “attraction factor” and therefore a threat. For this reason, special conditions were always established for regularization, which varied according to the different processes, having in common the requirement that undocumented people had to show their uninterrupted presence in Spanish territory and prove their “social roots” in the country.^8^ Therefore, there was a wide‐ranging consensus on the need of undocumented people to fulfil a series of conditions to access regularization, which took off the cards the notion of “papers for all” backed by migrant and pro‐rights associations. For this reason, a certain amount of undocumented people were always excluded.

In the case of #RegularizacionYa, this is the first time that arguments for regularization are not based on an instrumental view of migration (its utility), or on the calculation of the risks posed by a contingent of undocumented migrants. In this sense, it breaks a dynamic of middlemen in which experts in the field (e.g. academics) and administration managers counsel and legislate in the name of migrant interests. Hence, the general programme of #RegularisationNow has a highly symbolic importance. Focusing on the framework of rights and not on the windows of possibility, the platform broke with the pragmatism that prevailed in previous processes, which had generated high levels of tension among civil society organizations.^9^ In the pandemic context, the #RegularisationNow action strengthened alliances with different sectors of the Spanish civil society, including certain left‐wing political parties. These parties presented a non‐legislative motion in favour of migrant regularization in Parliament, in support of the campaign.^10^ Although this action highlights the strength that this migrant initiative acquired, we need to stress that non‐legislative motions have no prescriptive character and that the motion, although it was supported by eight of the ten parliamentary parties, obtained only a minority of votes (59 out of 350).

Nevertheless, it should be pointed out that these proposals do not limit themselves to maintaining traditional demands for migrant rights, taking over from the pro‐immigrant civil society mobilizations that monopolized the pro‐rights movement until 2005, with the last regularization process. On the contrary, they weave a creative bricolage of innovative demands, including using a specific language based on decolonial critique theories. This strategy signals a new path of political action in the framework of integration in a society that they defined as white, patriarchal and colonial. The manifesto that gave rise to the movement said explicitly:

As the articulated action within a statewide network of migrant collectives and self‐organised #antirracist people, we DEMAND the Government and all its competent authorities, the extraordinary, unconditional and indefinite regularisation of all migrants and asylum seekers/refugees in the face of the emergence of #covid19. Not leaving any person behind requires political will and a #regularisationnow^11^

In the discourse produced, we can record a peculiar use of the language, for instance, the adoption of the feminine as a generic universal. In Spanish grammar, the generic universal has been always masculine. Besides subverting the grammatical rule, this choice aligns with feminist positions that denounce gender bias in the Spanish language. One of the strategies by feminist critics directed at language is feminizing terms that are not officially recognized by the *Real Academia Española* (leading authority in linguistic norms), which always provokes strong criticism from both the linguistic officialdom and important sectors of society. This linguistic struggle is well known by the actors who join the #regularizacionYa! platform, which leads us to deduce that their choices are a conscious and deliberate bid to break the boundaries of both linguistic and political correctness, thus bringing the discourses of the pro‐immigrant movement up to the present. Furthermore, it represents a conscious effort to opt for a counter‐hegemonic discourse that challenges existing social relations, including gender relations.

Their discourse also assumes a holistic feature. Before COVID‐19, most of the actions and discourses of the pro‐immigrant movement had focused on the problems of migrants, understanding these as different from those of the Spanish majority. Nevertheless, in the pandemic context, both the discourses and actions of these migrant groups focus on the effect of neoliberal policies at the global and State levels, which affect the whole population. Even if they focus primarily on the impact of these policies on migrants, they always present it as an inherent part of Spanish society. In the founding manifesto, we can read:

The coronavirus pandemic has shown, once again, that it is the migrant and refugee population who suffer the most from the harmful effects of government policies of adjustments and cuts in the health, social, labour, and economic spheres. The consequence is the worsening of our living conditions, especially for the almost 600,000 people in an irregular administrative situation.

The relevance of self‐organization in a context in which migrant associations had always lagged behind Spanish NGOs is evident in the emphasis on discourses about the situation of vulnerability not being a social condition but the result of the violation of rights, which implies a direct criticism of the assistance‐based approach that has characterized the political action of pro‐migrant NGOs. One of the most significant steps of this network was the creation of mutual support networks to confront extreme vulnerability, such as the *Red Solidaria de Acogida de Lavapiés* ^12^ [The Network for Solidarity and Shelter in Lavapiés].

In the OSN campaigns, they have managed to involve famous artists, such as Paco León (actor) or Rozalén^13^ (singer‐songwriter) as well as renowned journalists like Mercedes Milá or Jordi Évole.^14^ The strength of this movement was also tested “offline”, during the demonstrations against racism organized in Spain after the assassination of George Floyd,^15^ where they played an important role. Furthermore, they displayed their strength supporting the non‐legislative proposal for regularization with mobilizations in front of Congress.^16^ Afterwards, the movement has continued to be very active, denouncing the situation of seasonal agricultural workers in the OSNs and on the streets and constituting the *Grupo de Acción Política de las Trabajadoras de Hogar* ^17^ (Domestic Workers’ Political Action Group), which brings together a variety of worker organizations at a national level. They also helped disseminate the campaign by the *Por Causa* initiative denouncing the “Migration Control Industry” costs in Spain and the benefits accrued by well‐known Spanish companies.^18^ Moreover, they support comprehensive local activities, collaborating with public and private entities in different programmes and events, always claiming their protagonist role as migrants and denouncing the forms in which they are made invisible or subordinated.

In all these campaigns and collective action, the main role falls almost exclusively on migrants, and especially women. However, it must be underlined that this is more the case in the on‐site activities (workshops, food distribution) than in the media communication, where gender parity is sought. In addition to the criticism of neoliberal capitalism, the messages produced by this network focus on denouncing racism and patriarchy. These three critical axes constitute the foundation of a movement’s political action that defines a host society shaped by a colonial alliance between patriarchy, racism and capital.

Concerning racism, the movement is very active in denouncing discrimination, police abuse, interpersonal racism or bureaucratic obstacles to regularization, and reporting the institutional racism of the migration policies of the Spanish State and the European Union. In the case of the Spanish State, the campaigns aimed, for instance, at rethinking and representing the colonization process of *Abya Yala* ^19^ (an Andean indigenous term for the American continent) both online and offline. Furthermore, the movement actively collaborates in the creation and dissemination of documentaries and news material produced by national and foreign media that reflect situations of abuse and exploitation in specific working sectors^20^ and denounce police campaigns based on racial profiling^21^ as well as the reception conditions of migrants arriving on Spanish coasts^22^ (and other European and American borders).^23^

## MIGRANT WOMEN STANDING OUT IN THE ASSOCIATIONS

The emphasis on the three axes mentioned above shows the vital role that migrant women play in the movement. These women are organized not exclusively but fundamentally in associations focused on the problem of working conditions. For instance, in a statement drafted by the collective action to denounce the situation of women day labourers in the Andalusian countryside, “Jornaleras en Lucha” (Women Day‐Labourers in Struggle) declared:

Spain, a state that extols its pact for "equality" and against gender violence, demonstrates once again how, from its structural racism, some lives are worth more than others; women, migrant, impoverished and dedicated to an undervalued productive sector such as the primary sector, are relegated to the background. The greatest difficulty in the fight against racism, sexual violence and the inhumane treatment of fellow Moroccan women day labourers in the red fruits [industry] in southern Spain, is to demonstrate that they are constantly subjected to these patterns of violence.^24^

Adding further:

We know that impunity is the existence of a structure of patriarchal, capitalist, colonial and racist oppression to silence the victims of the dominant system while granting privileges so that the subjects of power are exempted from any responsibility for their abuses and violence.^25^

On 23rd of March 2020, the association *Sindillar* (‘Home Workers Union’ in Catalan, @sindillar), one of the oldest in terms of their presence in the digital space, reinforced the support network they had contributed to create in the past and organized what they called a “resistance campaign” based on the manufacture of rag dolls named *sindirebels*. The raised money was allocated to women who had lost their jobs and the purchase of precautionary supplies for those who continued to work in unsafe conditions. This action was explained in the following terms:

This campaign is a way of keeping us more united than ever facing the crisis, which is affecting us especially because of our precariousness and the lack of response at the State level. Once again, we ask ourselves, who cares for those who care?

On 31st of March, their spokesperson, Rocío Echevarría, gave an interview to elperiodico.com in which she condemned the two options that remained for them during the lockdown: slavery, confinement of the worker in the home where she works, or dismissal. For her part, in this same article Carmen Juárez, spokesperson for the *Mujeres Migrantes Diversas* collective (@MujeresMigrante), stated that: “Among the more than 400 women that we know working as domestic and care assistants, only three are spending the lockdown at their home and with a guaranteed salary”.^26^

By 29th June, the accusations had turned into concrete demands: access to government subsidies for domestic workers. In May, the association released a documentary entitled “Care between lands. Who sustains life when women migrate”^27^ that narrates the lives of women working in care chains. For its part, the association SEDOAC (Active Domestic Service; *@sedoac*) was a fundamental pillar in the *#RegularizacionYa* action on the occasion of May Day and in the campaigns *#CuidaAQuienTeCuida* (#LookAfterWhoLooksAfterYou) and *#IMVSinNadieAtrás* (#IMVNoOneBehind). Its spokesperson, Edith Espinola, participated in a conversation with journalists, in which she denounced the conditions under which domestic workers had to work during the pandemic.^28^

The activity of these women is not limited to decrying the exploitative situations suffered by domestic workers, even if this was their main activity during lockdown and the launching of the regularization action at the national level. Before and after, they have developed fundamental trade union work. Thus, one of their most important stipulations is the demand for the application of the ILO (International Labour Organization) Convention 189 to domestic workers in 2011, entering negotiations with high‐level government representatives. However, their demands go beyond compliance with the Convention and aim to reformulate social relations to place care at the centre of people’s lives. In this respect, the boundary between domestic work and care work is a thorny issue. In the case of live‐in domestic workers, the boundary is completely blurred and requires careful and delicate negotiation. The associations referred are well aware of this.^29^

As pointed out, in addition to all of this, another crucial activity among these associations during confinement has been the participation in mutual support networks. In this regard, the association *Valiente Bangla* (Brave Bangla), created in 2007 to support the Bangladeshi community and other socially excluded people in the Autonomous Region of Madrid, in March 2020 organized a solidarity resistance cashbox linked with the food bank in the Lavapiés neighbourhood, where a large part of this community is based.^30^ At the same time, they undertook a campaign to raise awareness of the need for interpreters in hospitals during the pandemic’s peak. Their work was picked up by the media *El Salto*, which highlighted their capacity for networking.^31^

A fact that has to be taken into account is that both in the associations with a marked trade union purpose and in those more focused on assistance, the role of women is overwhelming, with one logical exception, the union of street vendors, since this is an almost exclusively male activity. Accordingly, it is essential to point out that mass migration has followed discernible patterns concerning the subject’s sex/gender systems. As Sassen points out:

In terms of their articulation in the mainstream global economy, the new economic literature on current globalisation proceeds as if this new economic phase is gender neutral, thereby rendering these gender dynamics invisible. This set of dynamics can be found in the alternative cross‐border circuits […] in which the role of women, and especially the condition of being a migrant woman, is critical (2000, pp. 507–508).

In a world where migration has become one of the most compelling societal challenges and where employment is a key dimension of citizenship, it is critical to stress that the integration of migrant workers is not only connected to the adaptation to the economic, social and cultural conditions, but also to the different forms of resistance against these circumstances. That is why in our digital ethnography we have witnessed how the claim actions of many associations move easily between the work and the neighbourhood of residence spheres. This is facilitated by migrants’ tendency to build dense, strong networks that, under certain circumstances, support unionization and political mobilization (Milkman, 2006). The centrality of care facilitates this transition between the different spheres of life within the dynamics of resistance. This is both the cause and consequence of the feminization of these networks. For example, on 29th April 2020, a Webinar took place organized by the Calala Foundation, the Oaxaca Consortium, IN‐Defensoras, the National Network of Women Human Rights Defenders of Honduras, the Network of Honduran Women Migrants and the Latina Network^32^ with the expressive title “Communalising emotions and struggles: reflections and practices of collective care for women defenders in the face of the current crisis”.^33^ Within these collectives, decolonial thinking is a central tenet of their discourse and proposals.

In this regard, it should be highlighted that the approaches and trajectories that we have been able to follow through the networks show an interesting variety of experiences and content among the associations, which are marked by their territorial origin and history and the biography of their members. Thus, there are associations that have been founded after a long trajectory of trade unionism in Spain by their founders, while there are other activists with previous political experience in their countries of origin.^34^ Most of them are women from Andean countries who came in the successive waves of visa exemptions that Spain established with countries in South America. Others come from Central America and the Caribbean (Honduras, El Salvador, Nicaragua, Cuba and Venezuela). Their migration is newer, and they have a high political profile and a solid attachment to decolonial approaches. On the other hand, Moroccan women are organized in defence of their rights as day labourers, and Bangladeshi women are fighting against language barriers and social exclusion.

But, beyond these differences, the strong interconnection and rich exchange of discourses and experiences are perceptible, both in the collective activities organized and in the discourse used to defend their demands, which confirms Milkman’s hypothesis of the strength and density of migrant networks, in this case, virtual networks.

## CONCLUSIONS

This paper demonstrates how the context of the pandemic in Spain has shown a growing trend, with roots in the previous years (Bermúdez et al., 2014; Cuberos Gallardo, 2009), towards a greater prominence and visibility in the political field for migrant associations, formal and informal, in which women are displaying a leading role. This mobilization was triggered by a situation of socio‐economic vulnerability generated by the COVID‐19 pandemic, which hit highly informal and precarious sectors, such domestic work, populated broadly by migrant women. During the Spring 2020 lockdown, a clear engagement of these women in the political arena emerged through formal and informal associations, generating solidarity networks with other civil society organizations within digital spaces.

As we observed in the digital ethnography, the tone of the discourses and the type of activities can be categorized within radical political positions. Even if they represent a political minority, their media impact has been significant at multiple levels. Locally, they have forced employers who do not comply with labour legislation to defend themselves against the complaints received. At the regional level, their reports provide information on good and bad practices in the different Autonomous Communities. At the State level, they have contributed decisively to strengthening labour inspection mechanisms and an increase in demands and fines, while their activities have been disseminated within activist networks globally.

They achieved this thanks to the clever use of social networks and a significant political capital accumulated through years of activism within pro‐migrant organizations. Indeed, although many of these networks' members are in an irregular administrative situation, most of the movement’s leaders have been forged in activism under the umbrella of prominent NGOs and trade unions. Some also had a background of political activism in countries of origin. Nevertheless, the pandemic has meant a qualitative leap in the leadership of these associations in terms of representativeness and visibility of their political and working rights, compared to the pre‐pandemic stage, when the main protagonists were pro‐migrant associations. During COVID‐19, they opted for self‐organization, converging in the interracial, interethnic, feminist and decolonial collective action #RegularizacionYa, generating socially creative responses through organizational bricolage (Castellani & Roca, 2022; Phillimore et al., 2019) with the resources accumulated in their networks and designing effective strategies of alliances among migrant associations and other civil society groups. In this sense, these associations are analogous to community unionism (Roca & Martín‐Díaz, 2017) making bricolage with elements from their cultural backgrounds and features of the host society in order to adapt to new circumstances. In pandemic times, this bricolage was based largely on the creation, consolidation and instrumental use of virtual social networks.

The prominent role played by migrant women in the politicization process experienced by many migrant associations during the pandemic is crucial for understanding this. The main associations defending labour rights in sectors such as domestic and care work, hospitality and agriculture in Spain are actually led by women and largely made up of women, although they are open to the participation of men working in domestic service and agriculture. Moreover, the political activation of migrant women within their organizations during the pandemic shows how the discourses and actions promoted through bricolage are permeated by care relations. Their actions often result in the reproduction of mutual support networks. The radical discourse produced by migrant organizations, the acquisition of their voice as well as the redefinition of relations with pro‐migrant civil society represent, in the end, an act of creative disruption (Stock, 2019) and resistance, in other words, an act of citizenship (Isin & Nielsen, 2008).

## CONFLICT OF INTERESTS

The authors declare that they have no conflict of interests.

## Data Availability

The data that support the findings of this study are available on request from the corresponding author. The data are not publicly available due to privacy or ethical restrictions.

## Endnotes

^1^
The first “state of alarm” in Spain was declared on the 15th of March 2020 (Royal Decree 463/2020 of 14 March 2020) and lasted until the 21st of June. This involved a general lockdown: all non‐essential activities were stopped.

^2^
Other campaigns were implemented by NGOs, for instance CEAR (Spanish Commission for Refugee Aid) with the campaign #Saludparatodos (#Healthforall).

^3^
Following Góralska (2020: 47), the digital space “can comprise anything that the web is made of – texts, videos, images, platform infrastructures, user behaviours, social relations, or an information network‐”.

^4^
The associations are @SEDOAC; @Sindillar; @AIPHYC1; @JornalerasL; @CPSproDerechos; @Valiente Bangla; @RMigradas; @FondoCalala; @CnaaeB; @CaravanaObrim; @RSAcogida; @porCausa.org.

^5^
Only since mid‐April 2020, 1 month after lockdown started, were (regular) domestic workers included in this group.

^6^
A BBC report documented this situation effectively https://www.youtube.com/watch?v=OVL4DOiuFt8 [retrieved on 08/06/2022].

^7^
This observation is based on Emma Martín Díaz’s direct experience in participating as a member of the panel of experts that advised the Spanish General Directorate of Migration in the regularization processes of 2001 and 2005.

^8^
The concept of “social roots” makes its appearance in the Organic Law 4/2000, article 31.3.

^9^
For instance, in the case of the migrant lockdown protest in Seville during the EU summit in June 2000, there was explicit conflict between the promoters of the initiative (immigrant associations supported by an anarchist trade union) and the Ombudsman’s Office and pro‐immigrant NGOs.

^10^
Letter to the Government #RegularizacionYa. regularizacionya.com.

^11^
Founding manifesto. https://regularizacionya.com [retrieved on 08/06/2022].

^12^
@RSAcogida.

^13^

https://www.youtube.com/watch?v=mf6ljZ9LSfM [retrieved on 08/06/2022].

^14^

https://www.youtube.com/watch?v=YvJZoEIMWpE [retrieved on 08/06/2022].

^15^
These demonstrations were widely reported in the press. Conservative media, however, emphasized the infringement of security measures in the pandemic. (El Mundo https://www.elmundo.es/madrid/2020/06/07/5edcc55721efa0d5268b4667.html [retrieved on 08/06/2022]).

^16^

https://www.youtube.com/watch?v=‐kveTp_NWg0 [retrieved on 08/06/2022].

^17^

https://labonne.org/sindillar‐encuentro‐estatal‐de‐accion‐politica [retrieved on 08/06/2022].

^18^

https://porcausa.org/industriacontrolmigratorio/ [retrieved on 08/06/2022].

^19^
Hashtag *#12DeOctubreNadaQueCelebrar*. https://www.youtube.com/watch?v=m085WRy6v7E [retrieved on 08/06/2022].

^20^
Documentary “*Después de las 8*”, Diario Público, premiere 22/11/2020. https://www.youtube.com/watch?v=ICiv644sgJ8 [retrieved on 08/06/2022].

^21^
It was precisely in June 2020 that the Twitter profile of the CNAAE, *Comunidad Negra Africana y Afrodescendiente en España* (Black African and African Descent community in Spain), was created. Their first tweets were aimed at denouncing the racial profiling of arrests by the law enforcement authorities.

^22^
#RutaCanaria.

^23^
@CoordinadoraOF.

^24^

https://regularizacionya.com/comunicado‐companeras‐jornaleras‐del‐campo/ [retrieved on 22‐06‐2022].

^25^
Ibid.

^26^

https://www.elperiodico.com/es/sociedad/20200331/empleadas‐hogar‐coronavirus‐7904816 [retrieved on 08/06/2022].

^27^
In Catalan “Cuidar entre terres. Qui sosté la vida quan les dones migren?” https://www.youtube.com/watch?v=2RM‐vlhl2xE [retrieved on 08/06/2022].

^28^

https://twitter.com/RegularizacionY/status/1270007021572689921?s=20 [retrieved on 08/06/2022].

^29^
This work and debate was well reflected in the Conference held at the University of Barcelona: “Beyond ILO Convention 189. Proposals and strategies of independent workers’ organisations for a decent regulation of domestic and care work in Spain”, in which Emma Martín participated.

^30^

https://twitter.com/RSAcogida/status/1270987189162295296?s=20 [retrieved on 08/06/2022].

^31^

https://www.elsaltodiario.com/rostroscuarentena/la‐asociacion‐valiente‐bangla‐y‐su‐autoorganizacion‐ante‐la‐crisis [retrieved on 08/06/2022].

^32^
All of these are feminist foundations and associations that promote the rights, participation and empowerment of women in Central America and Spain. The Calala Foundation has its origins in Spain, the Oaxaca Consortium in Mexico, IN.Defensoras in Nicaragua and the Red Latina is based in Latin America and the Caribbean.

^33^

https://twitter.com/laurarosillob/status/1255158589037129728?s=20, https://www.youtube.com/watch?v=H2lKwuQkR‐A [retrieved on 08/06/2022].

^34^
These data are drawn from informal conversations at in‐person events, phone interviews, DMs (private messages on Twitter) and conversations at shared events.
